# Recent Clinical Updates of Hypertrophic Cardiomyopathy and Future Therapeutic Strategies

**DOI:** 10.31083/RCM25132

**Published:** 2025-02-20

**Authors:** Mengya Zhao, Xianzhen He, Xinwen Min, Handong Yang, Wenwen Wu, Jixin Zhong, Hao Xu, Jun Chen

**Affiliations:** ^1^Sinopharm Dongfeng General Hospital (Hubei Clinical Research Center of Hypertension), Hubei University of Medicine, 442000 Shiyan, Hubei, China; ^2^Children’s Medical Center, Renmin Hospital, Hubei University of Medicine, 442000 Shiyan, Hubei, China; ^3^School of Public Health, Hubei University of Medicine, 442000 Shiyan, Hubei, China; ^4^Department of Rheumatology and Immunology, Tongji Hospital, Huazhong University of Science and Technology, 430030 Wuhan, Hubei, China; ^5^Shiyan Key Laboratory of Virology, Hubei University of Medicine, 442000 Shiyan, Hubei, China

**Keywords:** hypertrophic cardiomyopathy, pathogenesis, diagnostic, gene therapy, prognosis

## Abstract

Hypertrophic cardiomyopathy (HCM) is the most prevalent inherited cardiomyopathy transmitted in an autosomal dominant manner to offspring. It is characterized by unexplained asymmetrical hypertrophy primarily affecting the left ventricle and interventricular septum while potentially causing obstruction within the left ventricular outflow tract (LVOT). The clinical manifestations of HCM are diverse, ranging from asymptomatic to severe heart failure (HF) and sudden cardiac death. Most patients present with obvious symptoms of left ventricular outflow tract obstruction (LVOTO). The diagnosis of HCM mainly depends on echocardiography and other imaging examinations. In recent years, myosin inhibitors have undergone clinical trials and gene therapy, which is expected to become a new treatment for HCM, has been studied. This article summarizes recent clinical updates on the epidemiology, pathogenesis, diagnostic methods, treatment principles, and complication prevention and treatment of HCM, to provide new ideas for follow-up research.

## 1. Introduction

Hypertrophic cardiomyopathy (HCM) is a prevalent autosomal dominant genetic disease characterized by asymmetric ventricular hypertrophy and a small ventricular cavity. When diagnosing HCM, it is necessary to exclude myocardial hypertrophy caused by other cardiovascular diseases or systemic and metabolic diseases. Additionally, HCM is a significant contributor to sudden death among adolescents [1]. Patients can be divided into obstructive HCM (oHCM) and nonobstructive HCM according to the presence or absence of left ventricular outflow tract obstruction (LVOTO). The genetic characteristics of HCM can be divided into familial HCM and sporadic HCM. According to the location of myocardial hypertrophy, it can be divided into ventricular septal hypertrophy, apical hypertrophy, left ventricular diffuse hypertrophy, and biventricular wall hypertrophy. Since HCM can lead to a series of serious complications, including atrial fibrillation (AF) [2, 3], heart failure (HF) [4], and sudden death [5], it is imperative to understand the prevalence of HCM and investigate its associated complications [6, 7]. Many early studies reported an incidence of HCM of 1 in 500; however, in recent years, with the development of molecular genetic research and cardiac imaging, the detection rate of HCM has greatly improved, and the incidence of HCM has increased from 1 in 500 to 1 in 200 [8, 9]. The prevalence and morbidity of HCM in men are greater than those in women, and the mortality rate in men is lower than that in women, which has not been properly explained. However, some scholars have suggested that this may be related to the screening strategy used in this research, as well as genetics and sex hormones [10], and that there is little difference in the prevalence of HCM among different ethnic groups [8, 11, 12, 13].

## 2. Pathophysiology of HCM

Cardiomyocytes, fibroblasts, vascular smooth muscle cells, endothelial cells, and immune cells make up the various cell types discovered in the heart. Cardiomyocytes are permanent cells in the body, their quantity does not rise with age, but rather varies in size [14, 15, 16]. Hyperplasia of muscle fibers in cardiomyocytes leads to disordered arrangement of cardiomyocytes and irregular cell structure, resulting in a variety of clinical symptoms that affect the excitatory conduction function of cardiomyocytes and are associated with arrhythmias. Patients’ cardiomyocytes lose their normal contraction rhythm, which affects the heart’s normal contraction and contraction activities [17]. A study has demonstrated that HCM can impair cardiomyocyte metabolic function, causing changes in intracellular energy production and consumption [18].

The molecular and cellular mechanisms of HCM involve a variety of factors, including pathogenic variations in sarcomeric genes, noncoding genetic factors, and epigenetic and nongenetic factors; however, the etiology of HCM remains unknown in some patients.

Since 1989, when the β myosin heavy chain (*MYH7*) gene pathogenic variant was first identified as the cause of HCM, an increasing number of sarcomere genes have been shown to be associated with HCM, and more than 2000 mutations related to HCM have been reported [19, 20]. The common sarcomere genes associated with HCM include *MYH7*, myosin binding protein C (*MYBPC3*), cardiac troponin T (*TNNT2*), cardiac troponin I (*TNNI3*), regulatory myosin light chain (*MYL2*), essential myosin light chain (*MYL3*), α-tropomyosin (*TPM1*), and α-cardiac actin (*ACTC1*) [21, 22, 23]. The two most common sarcomere genes, *MYH7* and *MYBPC3*, encode the β-myosin heavy chain and myosin binding protein C, respectively [24, 25]. Sarcomere gene pathogenic variants in HCM account for approximately 20%~30% of HCMs [26, 27]. With increasing research, nonsarcomeric gene pathogenic variants have also been shown to be associated with HCM, and the nonsarcomeric genes identified thus far include α-actinin 2 (*ACTN2*), alpha-kinase 3 (*ALPK3*), cysteine and glycine rich protein 3 (*CSRP3*), formin homology 2 domain containing 3 (*FHOD3*), filamin C (*FLNC*), junctophilin 2 (*JPH2*), Kelch-like protein 24 (*KLHL24*), phospholamban (*PLN*), and ubiquitin E3 ligase tripartite motif protein 63 (*TRIM63*) [28]. Previous researchers reported that HCM patients with nonsarcomeric gene variants have a better overall prognosis and a lower probability of other cardiovascular complications than those with sarcomere gene variants [29, 30]. However, the current international HCM cohort shows no differences in terms of mortality, HF or sudden death between the genotype-positive and genotype-negative patients. These findings emphasize that genotype-negative patients do not have a better prognosis, nor can the results of genotypic testing provide a basis for patient management [27]. Clarifying the relationship between the underlying genetic etiology and the severity of the clinical phenotype in patients with nonsarcomeric gene variants is important. However, a large proportion of patients with HCM currently have no evidence of a genetic cause, approximately half of patients with HCM remain genotypically negative after genome-wide association studies (GWAS), and a large proportion of patients do not fit the Mendelian pattern of inheritance [31, 32]; thus, we conclude that HCM has a more complex genetic cause.

Although the etiological basis of HCM has not been fully defined, recent genetic studies have shown that alterations in the noncoding genome also play a large role in HCM. More than 90% of disease-related single nucleotide polymorphisms (SNPs) are located in the noncoding regions of genes, including regulatory regions such as promoters and enhancers [33, 34]. Variants in parts of the genome, such as introns, miRNAs, promotors/enhancers and long noncoding RNAs (lncRNAs), affect the overall structure, function, and expression of proteins to the same extent as coding mutations do [35].

Epigenetics refers to the alteration of gene expression without changes to the genetic code itself, which is achieved by the external modification of genes, including DNA methylation and demethylation, histone modification, noncoding RNA and posttranslational regulation. In patients with a family history of HCM, the expression of the phenotype can be influenced by genetic modifications. Exon methylation of the *MYBPC3* gene and histone deacetylases have been shown to be involved in the development of HCM [36]. However, the current epigenetic research on HCM is not mature, and more epigenomic research and HCM patient gene therapy experiments are needed to further improve this deficiency.

Being overweight or obese is usually associated with metabolic disorders such as blood sugar and lipids, which can affect the structure and function of the myocardium by changing myocardial energy metabolism and promoting inflammation and oxidative stress levels. Being overweight or obese may cause people with HCM to develop clinical symptoms at an earlier age and have a greater chance of developing hypertension and diabetes. A multicenter study of HCM revealed that obesity is independently associated with overall disease progression regardless of other known factors, with approximately 70% of patients with HCM being overweight, and obesity is associated with an increased likelihood of outflow tract obstruction, HF, and HF as determinants of adverse outcomes in patients with HCM [37]. Diabetes can lead to changes in myocardial fibrosis, steatosis and other metabolic factors, resulting in hyperglycemic heart disease. Diabetes is common in patients with HF, and is associated with morbidity and hospitalization rates. A Study has shown that diabetes is related to the clinical characteristics of HCM patients. HCM patients with diabetes have greater left ventricular hypertrophy and left ventricular enlargement on echocardiography and poor HF symptoms, exercise ability, New York Heart Association (NYHA) cardiac function classification, etc. In addition, patients with diabetes are more prone to AF and cardiac conduction disorders. The long-term outcomes and mortality associated with HCM are associated with increased risks of heart transplantation and sudden cardiac death in patients with diabetes [38].

Calcium (Ca^2+^) also plays an important role in cardiac contraction and relaxation, and studies have shown that pathogenic variants in myofilament proteins caused by HCM increase the sensitivity to Ca^2+^, leading to excessive cardiac contraction and promoting cardiac hypertrophy [39, 40, 41]. However, further research is needed to determine the molecular mechanism behind the increase in calcium ion sensitivity in HCM patients. The sodium (Na+) current also plays a very important role. An increase in the Na+ current in the cardiomyocytes of HCM patients is the basis of myocardial electrophysiological disorders. Some scholars have proposed that an increase in the Na+ current is the main reason for the increase in Ca^2+^ concentrations. In HCM patients, the L-type sodium current (INaL) is significantly increased in cardiomyocytes, resulting in an increase in sodium ion flow and an increase in the intracellular Na+ concentration during the action potential duration (APD). Moreover, along with the increase in the L-type calcium ion current (ICaL) and the decrease in K+, these changes are the basis for the prolongation of cardiomyocyte action potentials and arrhythmias in HCM patients. After intervention with the selective blocker ranolazine, the APD of cardiomyocytes was significantly shortened [42]. Ranolazine has been shown to reduce arrhythmias and diastolic dysfunction in patients with ischemic heart disease, and diazepam has a similar effect [43].

## 3. Diagnostic Approach 

### 3.1 Clinical Manifestations

Individual differences exist in the clinical presentation of HCM; some individuals may exhibit minimal symptoms or none at all, whereas others may experience more severe symptoms [13, 44]. Common clinical symptoms include dyspnea, palpitations, chest pain, syncope, arrhythmia, and even sudden cardiac death [45]. In patients with LVOTO, a pronounced systolic ejection murmur can be heard between the 3rd and 4th ribs of the left margin of the sternum, and there is often a transverse shift of apex beats in the precardiac area during palpations. For patients suspected of having HCM, a routine 12-lead electrocardiograph (ECG) remains an indispensable first step in evaluating HCM. A study has shown that ECG examination may be the only clinical manifestation in the early stage of HCM, and 90% of HCM patients have obvious ECG manifestations, often characterized by left ventricular hypertrophy, ST-T changes and pathological Q waves [46]. These clinical manifestations are mainly associated with increased ejection fraction due to increased cardiac systolic function and decreased stroke output due to diastolic dysfunction in patients. A study in *MYBPC3* and *MYH7* gene mutation-positive patients has compared initial and final echocardiography results. between the two groups of patients. Compared with *MYH7*-positive HCM patients, *MYBPC3*-positive patients are more prone to shrinkage function obstacles. There are fewer *MYBPC3*-positive patients at the onset of LVOTO, and the left ventricular ejection fraction (LVEF) is lower. Although HCM patients with both gene mutations present a slight decrease in left ventricular systolic function during follow-up, *MYBPC3*-positive patients had a higher rate of new-onset severe left ventricular systolic dysfunction. This study demonstrated that *MYBPC3* positivity, age, and AF are independent predictors of severe systolic dysfunction [47].

### 3.2 Imaging Examination

**Fig. 1.**
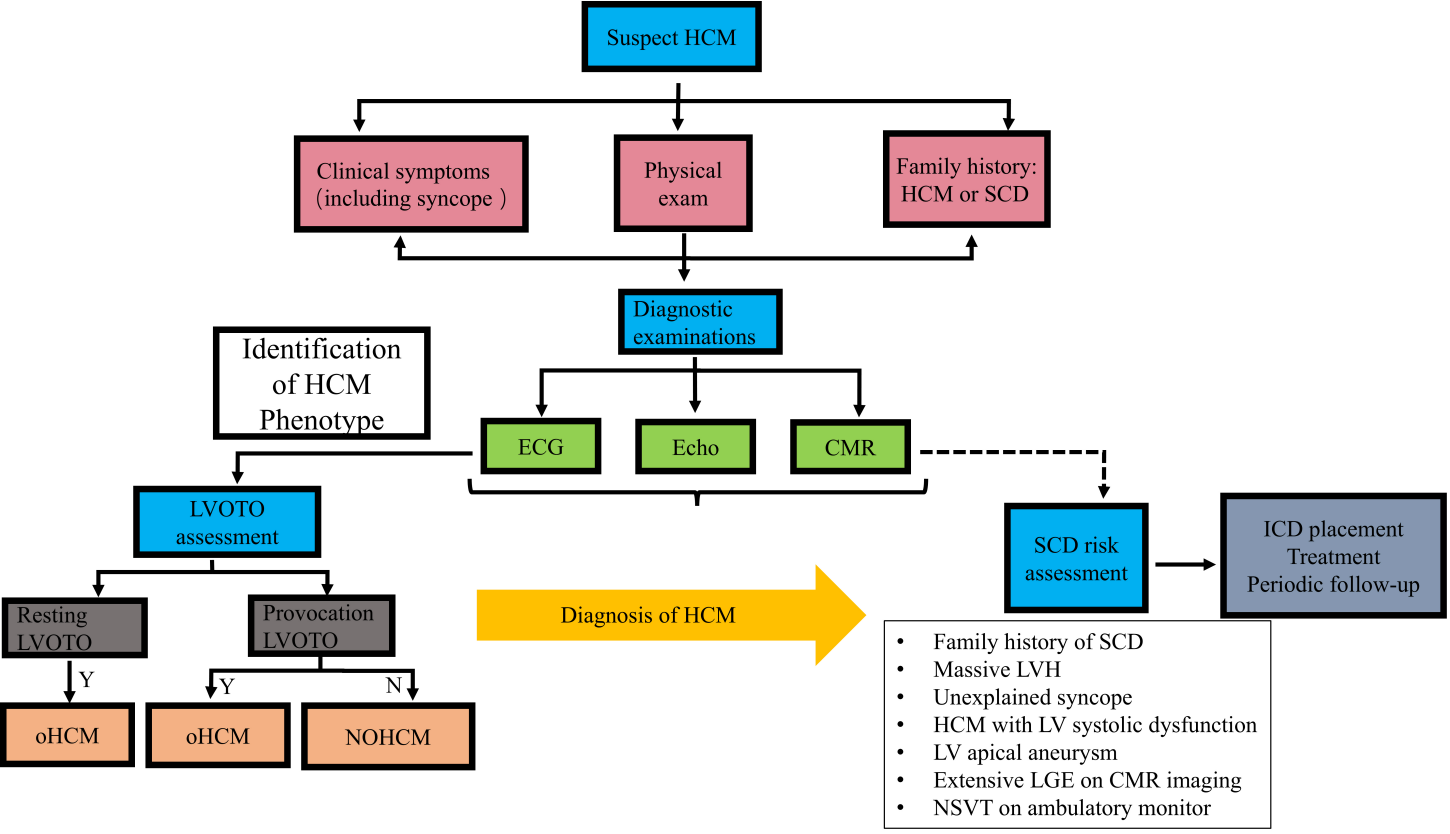
**The diagnosis flow chart of HCM**. HCM, hypertrophic cardiomyopathy; SCD, sudden cardiac death; ECG, electrocardiograph; Echo, echocardiogram; CMR, cardiovascular magnetic resonance; LVOTO, left ventricular outflow tract obstruction; Y, yes; N, no; oHCM, obstructive hypertrophic cardiomyopathy; NOHCM, non-obstructive hypertrophic cardiomyopathy; ICD, implantable cardioverter-defibrillator; LVH, left ventricular hypertrophy; LV, left ventricular; NSVT, non-sustained ventricular tachycardia; LGE, late gadolinium enhancement.

Imaging examinations play a central role in the diagnosis (Fig. 1), follow-up and prognosis of HCM patients. Echocardiography is an accurate and economical imaging method for the diagnosis of HCM that can help clinicians determine the degree of myocardial hypertrophy, evaluate the function and structure of the heart, detect whether the patient has heart valve abnormalities, and monitor the hemodynamics of the heart [48]. Through echocardiography, the degree of cardiac hypertrophy, abnormal ventricular wall motion, and changes in ventricular diastolic function, which are important features of HCM, can be observed. In addition, an echocardiogram can help rule out other heart conditions that may cause similar symptoms, such as hypertensive heart disease and myocarditis [48]. Consequently, echocardiography is crucial to the diagnosis and management of HCM because it allows medical professionals to identify lesions early on, gauge the severity of the condition, create a treatment strategy that works, and perform follow-up monitoring. Transesophageal echocardiography should also be performed if necessary. Perioperative transesophageal echocardiography should be performed before septal myectomy to determine the mechanism of LVOTO, evaluate the structure and function of the mitral valve, guide the formulation of surgical strategies, and evaluate the surgical effect and postoperative complications [49]. Unlike echocardiography, cardiac magnetic resonance (CMR) imaging accurately depicts the structure and function of the heart and, through the use of late gadolinium enhancement (LGE), can identify myocardial fibrosis. CMR is highly valuable for the clinical diagnosis of HF, the early detection of complications, risk assessment, and surgical plan formulation prior to myocardial resection [50]. The 2023 European Society of Cardiology (ESC) Guidelines for the treatment of cardiomyopathy fully affirm the importance of CMR in the diagnosis of cardiomyopathy. CMR combined with T1/T2 and LGE is highly valuable in the diagnosis of the cardiomyopathy phenotype, detection of disease progression, prognosis and risk stratification [51]. CMR is a powerful tool for the differential diagnosis of HCM and can effectively distinguish HCM from other diseases that may lead to increased myocardial thickness, such as physiological myocardial thickening in athletes, hypertensive cardiomyopathy, aortic valve disease, cardiac deposition disease, and mitochondrial cardiomyopathy [51]. CMR has the advantages of high spatial resolution, an unrestricted field of view, and strong visualization of tissue features, especially in identifying basal, apical and lateral hypertrophy, and apical aneurysms. The LGE technique is able to distinguish ischemic and nonischemic heart disease by identifying different enhancement patterns and is particularly effective in identifying local scarring and fibrosis of the heart muscle. A study has shown that there is a correlation between LGE and sudden cardiac death (SCD), and in the absence of other risk factors, patients with LGE enhancement have twice the risk of SCD as those without LGE. The presence and extent of LGE may be an independent prognostic factor in patients with sarcomeric HCM [52].

### 3.3 Laboratory Examinations

Blood parameters, liver and kidney function, electrolytes, and blood biochemistry, which can be used to assess the underlying condition of the patient, including whether there is damage to liver and kidney function, whether there is electrolyte disturbance, and whether there are other diseases, should be routinely used in the initial diagnosis of every HCM patient [53]. Biomarkers include brain natriuretic peptide (BNP) or N-terminal pro-brain natriuretic peptide (NT-proBNP), myocardial troponin or hypersensitive troponin. BNP and NT-proBNP can help to diagnose HCM complicated with HF and evaluate the progression and prognosis of the disease [54, 55]. Troponin is a sensitive myocardial marker, and studies have shown that elevated levels of hypersensitive troponin in HCM patients are correlated with disease severity and may be associated with multiple adverse cardiac events, which can provide a basis for the identification of complications such as HF and SCD [56, 57]. Laboratory tests are important for evaluating the degree of myocardial injury and HF in patients and for ruling out other cardiovascular diseases. Nonetheless, it should be noted that the diagnosis of HCM primarily depends on cardiac imaging tests such as cardiac ultrasonography, electrocardiogram, and cardiac magnetic resonance, in addition to a thorough assessment of the patient’s family history. As a supplemental method, laboratory testing can be used to assess a patient’s condition more thoroughly and create a treatment plan, which is crucial for assessing the condition and prognosis of HCM patients [58].

## 4. Treatment and Management

At present, there is no reliable specific drug to prevent or reverse the progression of HCM. The general principle of HCM treatment is to alleviate the symptoms of patients and reduce the occurrence of complications [53]. Different categories of HCM patients have different management objectives. For asymptomatic patients with nonobstructive HCM, if they have only local myocardial hypertrophy without hemodynamic abnormalities, they need both clinical observation and follow-up on a regular basis for risk stratification and prognostic evaluation of whether they have any complications. If complications are found, they should be treated. Beta-blockers can be added if tolerated [59]. In patients with nonobstructive HCM who have dyspnea or chest pain, the main purpose of management is to evaluate whether there are complications such as coronary heart disease, HF, arrhythmia, and AF and to actively treat these complications. Owing to the high mortality of patients with oHCM, active interventions, including drug therapy, interventional therapy, and surgery, are often needed [49].

### 4.1 Drug Therapy

As a first-line treatment for oHCM, β-blockers have been proven effective in many clinical trials. A study involving 29 patients with oHCM revealed that metoprolol relieved LVOTO at rest and during exercise, relieved symptoms, and improved quality of life in patients with oHCM [60]. A regional study including 251 patients with HCM revealed that early and sustained β-blocker intervention reduced HCM mortality, and mortality from complications such as HF was associated with the final dose of beta-blockers. This study also revealed that cardiac muscle removal and pacemaker implantation did not improve survival and that early maintenance of β-blocker therapy reduced mortality [61]. In addition, β-blockers have been found to prevent stress-induced arrhythmias in HCM patients [62]. These studies show that early β-blocker intervention can benefit patients with HCM. When patients cannot tolerate β-blockers, nondihydropyridine calcium channel blockers (CCBs), namely, verapamil or diltiazem, can be used [63]. This combination can be used in patients with hypertension [53]. For patients with oHCM and severe dyspnea at rest, hypotension, and LVOTO >100 mmHg, as well as for children <6 weeks of age, verapamil is potentially harmful [64]. If symptoms persist after the use of nondihydropyridine CCBs and β-blockers, disopyramide may be considered. Disopyramide has been demonstrated in numerous trials conducted in recent years to help people with HCM symptoms. A patient with oHCM who did not respond to medication therapy was described in a 2019 case study; nevertheless, the patient’s clinical symptoms considerably improved with the addition of disopyramide [65]. A small trial in the same year studied 41 patients with HCM and reported that patients with oHCM with a high ejection fraction had a significant decrease in LVOTO after receiving a relatively low dose of disopyramide; at the same time, the study suggested that patients with more severe disease had a poor response to treatment with disopyramide, and such patients should be considered for early surgical treatment [66]. Digoxin, angiotensin-converting enzyme inhibitors (ACEIs), angiotensin II receptor blockers (ARBs), dihydropyridine CCBs, and high-dose diuretics can aggravate obstruction and should be avoided [67]. For patients with obstructive HF with dyspnea, volume overload, or elevated left ventricular filling pressure, low-dose oral diuretics may be taken into consideration [49]. Drug Therapy of HCM is shown in Table 1.

**Table 1.** **Medical therapy in patients with left ventricular outflow tract obstruction (LVOTO ≥50 mmHg)**.

Drugs	Indications and characteristics
β-blockers	In the absence of contraindications, according to the heart rate and blood pressure, the dose was started at a low dose and gradually titrated to the maximum tolerated dose
Nondihydropyridine CCB (Verapamil/diltiazem)	β-blockers are ineffective or intolerant. For oHCM and severe dyspnea at rest, hypotension, LVOTO >100 mmHg, as well as children <6 weeks of age, verapamil is potentially harmful
β-blockers together with Verapamil/diltiazem	Can be used in patients with oHCM and hypertension
Disopyramide	For patients with oHCM who have persistent symptoms attributable to LVOTO despite β-blockers or nondihydropyridine CCB, can add a myosin inhibitor (adult patients only), or disopyramide, or SRT performed at experienced centers. Since disopyramide can enhance atrioventricular node conduction and has the potential to increase ventricular rate during AF episodes, it is recommended to use disopyramide in combination with β-blockers or verapamil/diltiazem
Mavacamten/Aficamten	Targeted on cardiac myosin ATPase reduce the formation of actin and myosin cross the bridge, so as to reduce the excessive contraction of cardiac muscle, improve the diastolic function. In patients with HCM who develop persistent systolic dysfunction (LVEF <50%), cardiac myosin inhibitors should be discontinued. In pregnant women, use of mavacamten is contraindicated due to its potential teratogenic effects
Small dose of diuretic	oHCM with dyspnea, capacity overload or left ventricular filling pressure is high, a cautious use of low-dose oral diuretics may be considered.
ACEI/ARB, dihydropyridine calcium antagonists, digoxin, high-dose diuretics	It can aggravate outflow tract obstruction and is not recommended

CCB, calcium channel blockers; oHCM, obstructive hypertrophic cardiomyopathy; SRT, septal reduction therapy; AF, atrial fibrillation; LVEF, left ventricular ejection fraction; ACEI, angiotensin-converting enzyme inhibitor; ARB, angiotensin II receptor blocker; ATP, adenosine triphosphatase.

### 4.2 Interventional Therapy and Surgery

Alcohol septal ablation (ASA) and septal myectomy (SM) have been extensively performed in recent years, and the observational literature from specialist centers with large surgical volumes has shown that patients with oHCM have excellent outcomes and that the operative and long-term mortality rates are low [68, 69, 70, 71, 72]. Patients with severe clinical symptoms such as dyspnea and chest pain even after medication treatment, NYHA cardiac function III/IV, or poorly managed left ventricular outflow tract gradient (LVOTG) may benefit from ASA and SM [73]. At present, there are many meta-analyses on radiofrequency ablation and ventricular septal cardiomyotomy. A 2017 study on surgical myectomy and ventricular septal ablation revealed that ASA and SM have their own advantages and disadvantages, and the choice of the two mainly depends on the technical level of medical institutions and the willingness of patients. In general, ASA is more common. SM, on the other hand, is carried out only in higher-level medical centers [74]. A 2020 study included all electronic databases as of February 2020 to compare the clinical outcomes of ASA and SM in oHCM patients and reported no significant differences in long-term mortality or stroke rates between the ASA and SM groups. The reduction in left ventricular outflow tract (LVOT) in the SM group was greater than that in the ASA group, but the improvement in clinical symptoms (NYHA grade and angina pectoris) was less, the ASA group had relatively few perioperative complications, and the rates of reintervention and pacemaker implantation were relatively high [75]. According to a 2022 study of adult and child HCM patients, SM in both adults and children reduced LVOT more than ASA did, but ASA was still a feasible method for patients at risk of heart surgery or who were more inclined to receive minimally invasive treatment [76]. A study of long-term mortality in ASA and SM patients in the same year revealed that patients receiving ASA had more complications when age, sex, and complications were consistent and that 10-year all-cause mortality was significantly greater than that in patients receiving SM; however, other factors contributing to death were not excluded [77]. According to numerous meta-analyses of SM and ASA, SM is significantly better than ASA in improving obstructions. In addition, for patients receiving SM and ASA, the mortality rate, complication rate, length of stay and cost of stay in high-volume hospitals are significantly lower than those in low-volume hospitals. Therefore, researchers suggest that patients with oHCM should be referred to high-volume centers for treatment, which can improve the hospitalization outcomes of patients [78].

With the advent of M-mode ultrasound technology, mitral valve structural abnormalities have been found to play an important role in the occurrence and development of HCM. Systolic anterior motion (SAM) caused by abnormal mitral valve structure is an important factor for LVOTO in HCM patients. Surgical repair of mitral valve malformation is, therefore, becoming increasingly important. Patients with mitral valve malformation should receive not only ventricular septal reduction treatment but also mitral valve repair joint muscle resection. Mitral valve abnormalities in oHCM patients include leaflet overlength and various abnormalities of the valve, such as papillary muscle and chordae tendineae malformations, which can be visualized by transthoracic echocardiography, transesophageal echocardiography, and cardiac magnetic resonance imaging [79].

### 4.3 Lifestyle Changes

Studies have shown that moderate intensity exercise can improve symptoms in patients, and previous small animal experiments have shown that exercise reduces cardiac hypertrophy and myocardial fibrosis [80, 81], but large randomized controlled trials are needed to confirm these findings. When patients with HCM receive exercise training, they need to be closely guided by experts and undergo a detailed risk assessment. Exercise training is not recommended for patients with weight and metabolic increases and excessive psychological stress. Patients with HCM should consume a balanced diet, mainly a low-salt, low-fat and high-fiber diet; quit smoking; limit alcohol consumption; and undergo regular psychological screening to avoid anxiety [82]. In general, the daily work of most patients with HCM is not affected, and a comprehensive clinical assessment is required to determine whether they can engage in work requiring heavy physical strength or high physical strength. For women with HCM, both patients and their families should receive systematic genetic counseling related to HCM before pregnancy. A multicenter prospective study suggested that most women with HCM have good tolerance for pregnancy, but cardiovascular complications are not uncommon [83]. Cardiac volume overload during pregnancy can cause ventricular dilation, relieve symptoms of outflow tract obstruction, and improve diastolic function in patients; thus, pregnancy termination is not recommended in HCM patients without adverse cardiovascular events. A study of pregnant HCM patients with serious adverse cardiovascular events such as AF, nonsustained ventricular tachycardia, cardiac arrest, and acute left HF during follow-up revealed that sarcomere pathogenic variations were present [84]. However, the association between sarcomere gene variations and overall prognosis remains controversial. To reduce the risk of cardiovascular events during pregnancy, female patients should be rigorously consulted before becoming pregnant. They should also receive close monitoring and the best care possible during and after pregnancy [85, 86, 87].

## 5. Complications and Prognosis

HCM frequently results in arrhythmias, cardiac failure, thrombosis, and abrupt death. The most prevalent arrhythmia associated with HCM is AF. Research indicates that AF is caused by aberrant mechanical and electrical remodeling of the heart, which is caused by changes in the tissue structure of the myocardium and is frequently associated with a poor prognosis [3]. Recent studies suggest that left atrial (LA) myopathy plays an important role in the development of AF [88]. The incidence of AF in HCM patients increases with increasing disease severity. In HCM patients, factors such as ventricular remodeling, diastolic dysfunction, mitral regurgitation, and LVOTO lead to increased left atrial load, leading to adverse remodeling and dysfunction. In addition, cardiomyocyte hypertrophy, fibrosis, and microvascular dysfunction also increase the load on the left atrium. These factors lead to impaired left atrial function and are prone to AF. AF and left atrial disease significantly affect the prognosis of patients with HCM, increasing the risk of HF and embolic stroke. Therefore, for patients with HCM, early identification and management of left atrial lesions and AF are critical [88]. To investigate the relationship between stable sinus rhythm and the incidence of stroke in HCM patients, patients were divided into a stable sinus rhythm group, a preexisting AF group and a new AF group. The incidence of stroke was greater in HCM patients with stable sinus rhythm and was associated with the occurrence of AF, and severe LA dilation was a strong predictor of stroke, further emphasizing the efficacy of anticoagulation therapy in HCM patients with AF [89]. A study has indicated that the incidence of thromboembolism in patients with AF who are not receiving anticoagulation is more than seven times greater than that in patients receiving regular anticoagulation, and AF is the most common cause of thromboembolism in patients with HCM [90]. HFis a frequent side effect of end-stage HCM. The majority of patients initially have HF with preserved ejection fraction (HFpEF), which is associated with a worse prognosis than patients without HF. It has been observed that 43.5% of HCM patients can develop HFpEF, and the findings demonstrated increased ventricular wall thickness, increased LVOT pressure differential, increased proportion of AF, increased incidence of all-cause mortality, and increased incidence of cardiovascular death [91]. For patients with advanced HF in HCM, heart transplantation is often the best option [92, 93]. An implantable cardioverter-defibrillator (ICD) is now the most successful method of preventing sudden death in patients with HCM [49]. Further research revealed an inverse U-shaped association between projected SCD risk and left ventricular (LV) hypertrophy in young HCM patients, suggesting that anticipated SCD risk may start to decrease and that increases in LV hypertrophy above a certain threshold are not linked to extra risk [94]. The 2023 ESC Guidelines for the treatment of cardiomyopathy developed the SCD Risk Prediction tool to assess the risk of SCD in patients with HCM regularly. The factors associated with the HCM risk-SCD score include age, unexplained syncope, LVOT pressure stage, maximum ventricular wall thickness, left atrial size, nonsustained ventricular tachycardia (NSVT), family history of SCD, left ventricular systolic function, and degree of myocardial scarring. ICD implantation is recommended for primary prevention in patients with high-risk HCM risk-SCD scores (5-year SCD risk >6%). Secondary prevention is recommended for patients with cardiac arrest due to ventricular tachycardia or ventricular fibrillation, syncope due to spontaneous persistent ventricular tachycardia, or hemodynamic instability, and a life expectancy of 1 year [51]. A study that followed the efficacy of HCM patients with ICD implantation found that only patients with strict ICD indications experienced major cardiovascular events and that the associated complication rates were similar among all ICD implants, whereas the incidence of long-term adverse events was greater among those with ICD implants [95]. The American College of Cardiology/American Heart Association (ACC/AHA) intensive strategy for preventing sudden cardiac death in high-risk HCM patients was able to prospectively identify 95% of patients with potentially fatal ventricular tachyarrhythmias and was proven to be superior to the ESC risk score [96]. Syncope is a risk factor for SCD and may affect the quality of life and morbidity of HCM patients. At present, the cause of syncope in HCM patients is not clear, and the management of syncope is affected mainly by the SCD risk score. Therefore, a study is still needed to explain the mechanism of syncope in HCM patients [97]. Although each case of HCM has a different prognosis, people with this condition can benefit from early detection and treatment, which also lowers the risk of complications. After receiving active therapy, the majority of confirmed patients remain the same as normal individuals do; however, patients who experience severe complications typically have a dismal prognosis [98]. Therefore, to continuously track changes in symptoms, LVOTO, left ventricular function, and the occurrence of additional adverse events, customized lifelong follow-up plans on the basis of illness severity, age, and sex are needed.

## 6. Future Research Directions

With the steady maturation of HCM genetic research in recent years, genetic testing has identified apparent harmful genes in approximately 20%–30% of HCM patients, and the final clinical phenotype of HCM is the result of the interaction of genotypes, modifiers, environmental conditions, and other factors. Furthermore, it is still unknown what causes and how some HCM patients develop [99]. Gene therapy treats cardiomyopathy by introducing new genes or modifying existing genes and their regulatory moieties. At present, some progress has been made in clinical trials of gene therapy, but the dose-dependent immune response is still the main obstacle to its application. For Duchenne muscular dystrophy, gene therapy has been approved. Clinical trials of gene therapy for rare cardiomyopathies, such as Danon and Fabry disease, are also underway. In HCM and arrhythmia animal models, gene therapy also shows promising results. In the future, the goals of gene therapy include the development of safer and more effective delivery carriers, as well as specific cardiomyopathy gene targets, to improve the effectiveness of gene therapy in humans [100]. Gene therapy may be a feasible treatment method for HCM patients, but the design of gene therapy regimens still faces many challenges due to the inconsistency between the pathogenic type and genotype of HCM patients [101]. Many studies on long noncoding RNAs (lncRNAs) have emphasized their important role in heart development. Moreover, lncRNAs are involved in the occurrence of various cardiovascular diseases. Some scholars have proposed that the role of lncRNAs in the prevention and reversal of cardiac hypertrophy, including ischemic injury, cardiac hypertrophy, and myocardial fibrosis, which promote cardiac remodeling, can provide a basis for the design of future gene therapies [102, 103, 104].

Myosin inhibitors have emerged as a new therapy option for HCM in recent years [105, 106]. Mavacamten was the first myosin inhibitor developed in recent years. By targeting myocardial myosin ATPase, Mavacamten can reduce the formation of an act-myosin cross bridge, alleviate myocardial hypercontraction, improve diastolic function, and lower LVOTG. Mavacamten’s ability to improve HCM symptoms has been extensively demonstrated [107, 108, 109, 110, 111, 112]. Aficamten (also known as CK-274), the second myosin inhibitor to enter clinical trials, has been shown to have a shorter half-life and a wider therapeutic window than Mavacamten does; in patients with symptomatic oHCM, it can significantly improve the patient’s peak oxygen uptake rate and is currently undergoing phase III clinical trials [113, 114, 115]. According to the latest 2024 AHA/ACC/AMSSM/HRS/PACES/SCMR guidelines for the management of HCM, in patients with HCM who develop persistent systolic dysfunction (LVEF <50%), cardiac myosin inhibitors should be discontinued. In pregnant women, the use of Mavacamten is contraindicated because of its potential teratogenic effects [64].

With the progressive development of cardiac regenerative medicine, researchers have shifted from regarding the heart as an organ that cannot be regenerated to recognizing that intercellular communication between heart cells has enormous potential for the treatment of heart diseases [116]. According to a study, in addition to the renewal of cardiomyocytes, the correction of the cardiac microenvironment and vascular targeted therapies and immunotherapy are emerging concepts for reversing heart diseases. Intercellular communication between cardiac cells is required for heart repair and regeneration and it can be targeted therapeutically via a variety of techniques. These treatments involve inhibiting cardiac fibroblast activation, depleting certain activated fibroblast subsets, and inducing a functionally active vascular system [117].

## 7. Conclusions

HCM is a common genetic cardiomyopathy with a prevalence as high as 1/200 and is the most common cause of sudden death in adolescents. However, there are still no specific drugs that can radically resolve the clinical symptoms caused by myocardial hypertrophy. Gene therapy can act on the initial changes in the pathophysiology of HCM, which is a highly promising treatment. In the future, extensive genetic studies in many related patients are still needed to fully understand the associations between the genotypes and phenotypes of different patients, and to provide more effective treatment strategies for patients with HCM.
